# Development, Validation, and Application of the LC-MS/MS Method for Determination of 4-Acetamidobenzoic Acid in Pharmacokinetic Pilot Studies in Pigs

**DOI:** 10.3390/molecules26154437

**Published:** 2021-07-23

**Authors:** Paulina Markowska, Zbigniew Procajło, Joanna Wolska, Jerzy Jan Jaroszewski, Hubert Ziółkowski

**Affiliations:** 1 Department of Pharmacology and Toxicology, Faculty of Veterinary Medicine, University of Warmia and Mazury in Olsztyn, Oczapowskiego 13, 10-718 Olsztyn, Poland; paulina.markowska@uwm.edu.pl (P.M.); jerzyj@uwm.edu.pl (J.J.J.); 2 Department of Epizootiology, Faculty of Veterinary Medicine, University of Warmia and Mazury in Olsztyn, Oczapowskiego 13, 10-718 Olsztyn, Poland; zbigniew.procajlo@uwm.edu.pl; 3 Department of Anaesthesiology and Intensive Care, Faculty of Medicine, Collegium Medicum, University of Warmia and Mazury in Olsztyn, Al. Warszawska 30, 11-082 Olsztyn, Poland; joanna.wolska@uwm.edu.pl

**Keywords:** 4-acetamidobenzoic acid, validation, pharmacokinetic, pigs, LC-MS/MS

## Abstract

Each drug has pharmacokinetics that must be defined for the substance to be used in humans and animals. Currently, one of the basic analytical tools for pharmacokinetics studies is high-performance liquid chromatography coupled with mass spectrometry. For this analytical method to be fully reliable, it must be properly validated. Therefore, the aims of this study were to develop and validate a novel analytical method for 4-acetamidobenzoic acid, a component of the antiviral and immunostimulatory drug Inosine Pranobex, and to apply the method in the first pharmacokinetics study of 4-acetamidobenzoic acid in pigs after oral administration. Inosine Pranobex was administered under farm conditions to pigs via drinking water 2 h after morning feeding at doses of 20, 40, and 80 mg/kg. For sample preparation, we used liquid–liquid extraction with only one step—protein precipitation with 1 mL of acetonitrile. As an internal standard, we used deuterium labeled 4-acetamidobenzoic acid. The results indicate that the described method is replicable, linear (r^2^ ≥ 0.99), precise (2.11% to 13.81%), accurate (89% to 98.57%), selective, and sensitive (limit of quantitation = 10 ng/mL). As sample preparation requires only one step, the method is simple, effective, cheap, and rapid. The results of the pilot pharmacokinetics study indicate that the compound is quickly eliminated (elimination half-life from 0.85 to 1.42 h) and rapidly absorbed (absorption half-life from 0.36 to 2.57 h), and that its absorption increases exponentially as the dose is increased.

## 1. Introduction

4-acetamidobenzoic acid (PAcBA) is one of the naturally occurring, acetylated metabolites of 4-aminobenzoic acid [1] and is a component of the antiviral and immunostimulatory drug Inosine Pranobex (Inosiplex; Isoprinosine; Methisoprinol) [2,3,4,5]. Because of the increasing level of bacterial resistance to antimicrobials and the limitations in direct anti-viral therapy, an attempt has been made to improve immune defense mechanisms by searching for potential new drugs that stimulate the immune system [6,7,8]. For this reason, Inosine Pranobex is a potential candidate for preventive and/or therapeutic purposes, especially as an antimicrobial alternative in veterinary medicine. 

However, although Inosine Pranobex shows antiviral and immunostimulatory effects under in vitro conditions, it is unclear how this translates to in vivo conditions in animals. This is because, as with every drug, it undergoes pharmacokinetic (PK) processes in the body such as absorption, distribution, metabolism, and elimination [2]. To test the real in vivo effect of this drug (its pharmacodynamic effect), it is crucial to know its PK based on the quantitation of concentrations using a validated analytical method, which has not been performed in animals. 

Additionally, due to the fact that Inosine Pranobex consists of three different components, the PK has to be determined for each component. So far, the PK of PAcBA has only been examined in humans [9,10,11]. That study did not account for a potential matrix effect, and it used HPLC with UV light detection, which is less sensitive than tandem mass spectrometry. Moreover, tandem mass spectrometry allows the use of an isotopic standard to ensure more consistent results. 

Therefore, the objectives of the present study were to develop a novel method that uses tandem mass spectrometry for PAcBA analysis and to validate it with plasma from humans and 12 animal species, including testing for potential matrix effects. The method was subsequently applied in the first PK study of PAcBA in pigs after oral administration of three doses of Inosine Pranobex under farm conditions.

## 2. Results and Discussion

No papers on PAcBA analyses conducted with HPLC-MS/MS could be found in the available databases. Therefore, a new method for analysis of this drug was developed via the following sequence of steps: first, the most suitable detector parameters were chosen; second, the appropriate chromatographic conditions were selected; third, a method of quick, short, effective, easy, and cheap analyte extraction was developed; and fourth, the method was validated with human and animal plasma. Once developed and validated, the method was subsequently applied in a pilot study to determine the PK of PAcBA following oral administration of Inosine Pranobex to pigs at three different doses.

### 2.1. LC-MS/MS Parameters

The molecular weight of PAcBA is 179.175 g/mol and that of the internal standard (IS) is 182.193 g/mol. On this basis, the parent ions of the molecules were sought, assuming that each substance is ionized only once (*m/z* = 1/1) within the nitrogen atom. A thorough analysis of the PAcBA and IS mass spectra obtained by MS/MS operating in positive electrospray mode gave an *m/z* ratio of 180.20 for PAcBA, and 183.20 for the IS. Subsequently, precursor ion fragmentation was conducted, and the product ions were identified, with the best results achieved for particles with m/z values of 94.0 and 95.0 for PAcBA and IS, respectively. For each analyte, one transition was measured. The detailed parameters of HPLC-MS/MS are summarized in Table 1.

### 2.2. Chromatographic Conditions

The first step toward establishing the chromatographic conditions was the choice of a suitable column. This choice was affected by the properties of PAcBA, which is a relatively polar compound (XLogP3 1.3, Hydrogen Bond Donors—2, Hydrogen Bond Acceptors—3). A 150 × 3 mm Atlantis T3 analytical column with a 3 µm particle size was used in the analyses; however, a 150 × 3 mm XBridge column with a 3.5 µm particle size was also suitable for studying these analytes (Supplementary Figure S1). The method developed by Chen et al. 2013 for PAcBA analysis employed a universal C18 column, with a diameter and particle size much larger than those in the column used in this experiment [11]. Moreover, the Atlantis T3 column retains polar compounds better than the C18 column, which is why the former was considered a better option. Additionally, the optimum LC chromatographic conditions were determined, such as the mobile phase composition, gradient, flow rate, and temperature (Table 1). The initial phase comprised 0.2% formic acid (FA) in water with 0.2% FA in acetonitrile (ACN) at a ratio of 9:1 *v/v*, and the temperature was set at 35 °C. However, in these conditions, PAcBA separation proved unsatisfactory because of interference from unidentified background noise, and the shape of the IS peak was asymmetrical at the top (Supplementary Figure S2). For this reason, the separation was modified by increasing the ratio of 0.2% FA in water to 0.2% FA in ACN. The best result was achieved with a ratio of 99:1 *v/v* when the gradient started with the column temperature set at 20 °C (Table 1). For comparison, Chen et al., 2013 used a mobile phase composed of methanol—0.2% ammonium acetate and a 0.2% acetic acid solution at a ratio of 15:85 *v/v* [11]. We rejected the use of methanol due to maximal reduction of the baseline during analysis.

### 2.3. Development of Sample Preparation

**Figure 1:**
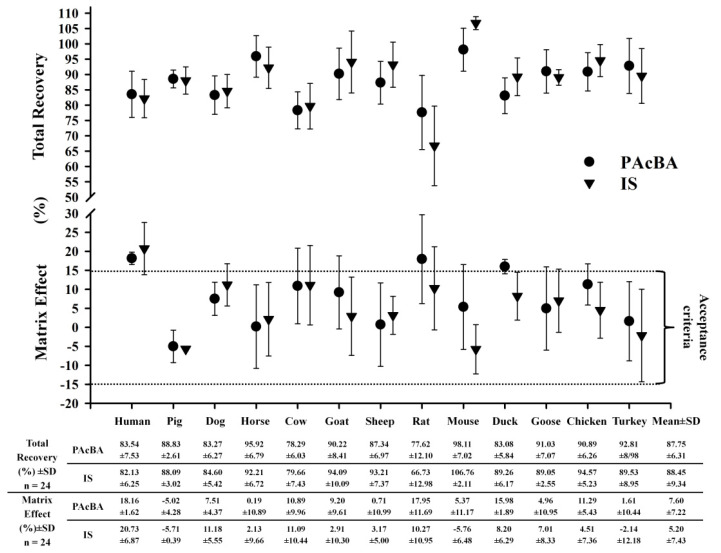
Matrix effect and total recovery of 4-acetamidobenzoic acid (PAcBA) and internal standard (IS) in humans and twelve animal species. Each point represents the mean value (±SD) calculated from six replicates of four quality control points for each matrix and reference sample (*n* = 24 per point).

The preparation of a sample for analysis is the main element in the development of a new analytical method for a given compound because, to ensure reproducibility, the validation of the whole method focuses mainly on the way the sample is prepared [12,13]. In the literature, only one publication by Chen et al., 2013 could be found regarding the preparation of the matrix, which was plasma (for the extraction of PAcBA) [11]; thus, it was decided to develop a new method for the preparation of plasma samples for the determination of PAcBA. As the main goal was to obtain a quick, easy, short, sensitive, and low-cost method of plasma purification, it was decided to use the liquid–liquid extraction (LLE) technique despite its disadvantages. As organic solutions for the purification of samples are most commonly used in LLE techniques, 1 mL of ACN was used for protein precipitation. For the extraction procedure, 1.5 mL of 1,2-dichloroethane or ethyl acetate was used. In contrast, Chen et al., 2013 used an LLE extraction procedure with hydrochloric acid for acidification and ethyl acetate as an extractant [11]. However, as they used UV detection in their analytical method, they might not have noticed potential problems with the matrix effect. The results of the present study were surprising; despite the fact that the samples were only treated by denaturation with ACN, the recovery was over 80%, and the matrix effect remained on an acceptable level, as shown by the validation protocol (Figure 1). However, in the samples extracted with 1,2-dichloroethane or ethyl acetate, even though the recovery was higher, the matrix effect was also higher (Supplementary Table S1). An advantage of the method presented here is that, because MS is used for detection, a stable isotopically labeled (SIL) analog of PAcBA can be used as an IS. In contrast, the method presented by Chen et al., 2013 used a UV detector, which meant that paracetamol was used as an IS instead of a SIL [11]. In such a situation, the extraction procedure may be much more difficult than simple protein precipitation. Thus, even though protein precipitation is not the most effective method of purifying the sample, based on the results obtained from the validation protocol in the present study and the fact that it allows more rapid utilization of the column, it was decided to use it for validation and subsequent analysis of pilot tests.

### 2.4. Validation

The method presented here was fully validated using pig plasma. Additionally, total recovery and matrix effect tests were also carried out with blood drawn from humans and eleven animal species. 

The values of “r” and “r^2^” for the linear regressions with the data from calibration were above 0.99, which met the acceptance criteria for linearity (Table 2). Additionally, because the literature contains no information on expected concentrations of PAcBA in pig blood [12], the range of the curve was expanded as far as possible, with a 1000× difference between the concentrations at the first and the last points on the curve. Throughout this expanded range, all acceptance criteria were met (Table 2, Supplementary Table S2). 

The accuracy and precision were estimated for all quality controls (QC) and the lowest limit of quantitation (LLOQ), both for the preparation of a single sample and for the preparation of six samples over a period of three days. The intra-day precision for all QC and the LLOQ was 2.11% to 13.81%, and the accuracy was 1.43% to 11.0%. The inter-day precision for all QC and the LLOQ was 3.43% to 10.93%, and the accuracy was 2.7% to 8.78% (Table 3, Supplementary Table S3).

The limit of detection (LOD) was set at 3.27 ng/mL ± 1.48 (a signal-to-noise ratio S/N not lower than 3:1) and the LLOQ was 10.0 ng/mL ± 1.09 (S/N = 15.69 ± 3.95) (Table 3, Supplementary Table S4, Supplementary Figure S3). As the samples for analysis were diluted four times for sample preparation during protein precipitation, the use of this method in such conditions did not allow very low PAcBA levels (<3 ng/mL) to be determined, which may be a limitation when investigating endogenous PAcBA in animals.

The selectivity/specificity of the method showed no significant peaks during PAcBA and IS retention in drug-free plasma obtained from blood collected from clinically healthy human and animals, and there was no significant carryover of PAcBA and IS during the analysis of high concentrations of these analytes (Table 3, Supplementary Table S5). However, it should be noted that there were several species (Supplementary Figure S4) for which small peaks appeared with an S/N lower than 10:1, which could indicate the presence of endogenous PAcBA. This phenomenon should be monitored by blank sample analyses in each test because of the risk that an endogenous analyte could appear that could be of particular importance in an attempt to optimize the method for the investigation of concentrations much lower than those possible with the method used in this experiment. 

In the method presented here, the mean total recovery for all species was 87.75% ± 6.31% for PAcBA and 88.45% ± 9.34% for IS (Figure 1). Both analytes appeared to be stable after 3 h at the sample processing temperature, after 48 h in an autosampler at 4 °C, after 1680 h of thawing and freezing cycles, and for 120 h as a prepared working standard stored in a refrigerator (2 °C) (Table 4). 

Endogenous PAcBA signals were not found in the matrices of the examined species (Supplementary Figure S3). The mean matrix effect for all species was 7.60% ± 7.22% for PAcBA and 5.20% ± 7.43% for IS, although in humans, ducks, and rats, the matrix effect was above the acceptable limit (Figure 1). As this method of sample purification is the “dirtiest” sample preparation technique, attention should be paid to each matrix used for the analysis, as the presence of endogenous PAcBA probably varies from individual to individual and may change depending on the matrix origin (Supplementary Figure S4).

### 2.5. Pharmacokinetics

**Figure 2:**
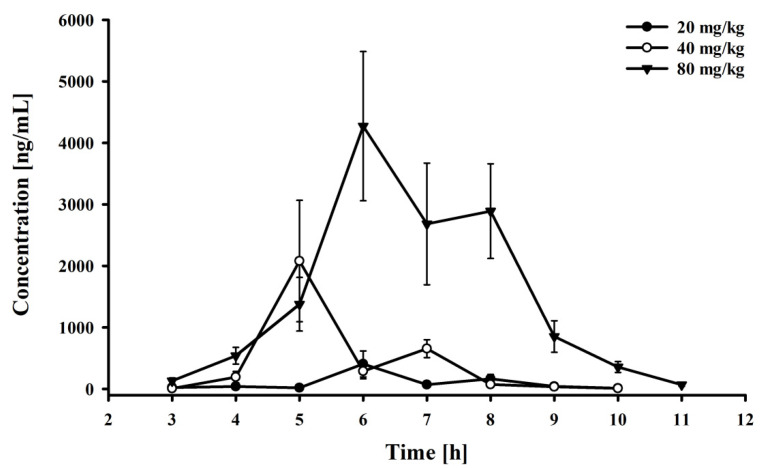
Plasma-concentration–time profile of 4-acetamidobenzoic acid after oral administration to pigs at a different doses.

The experiment demonstrated that the drug was absorbed relatively quickly in pigs (Figure 2; Table 5), although the mean absorption time (MAT) and half-life in the absorption phase (t_1/2kab_) differed somewhat between groups (Table 5). These differences, as well as the delayed absorption time, most likely resulted from the way the drug was administered (farm conditions). The animals received the drug in water 2 h after feeding. As they had probably drunk water when being fed, they most likely began to consume the drug some time after it was provided to them. The present results are consistent with those on the PK of Inosine Pranobex that are available in the literature; in studies of both Rhesus Monkeys [14] and healthy volunteers [11], PAcBA was also rapidly absorbed. Interestingly, in the present study, the AUC_(0→t)_ increased around 3.65 times despite the drug being administered in water for several hours with each doubling of the dose. The best fit to this data was obtained with power regression; the equation was y = 2.6707x^1.9367^, with r^2^ = 0.99995 (Supplementary Figure S5). In contrast, the best fit to the change in the values of C_max_ was obtained with logarithmic regression (y = 2788.3976 ln(x) − 8033.1062; r^2^ = 0.994). Although the precise determination of these parameters is complicated by the limitations associated with administration of the drug and the number of animals, these results indicate that changes in PAcBA concentration in plasma clearly reflect changes in drug dose. 

In the study presented here, the apparent volume of distribution without bioavailability correction (Vd_area_/F) was high, similar to what was observed by Chen et al., 2013 [11]. This result can be explained by several phenomena: either the drug absorbed poorly, or it was subject to efficient, rapid metabolism and/or rapid elimination. Streeter and Pfadenhayer 1984 suggested that the bioavailability of PAcBA is high, as well as the metabolism of the compound [14]. However, in the present study, these phenomena were difficult to assess. Nevertheless, the half-life in the elimination phase (t_1/2kel_) and MRT values of PAcBA (Table 5), as well as the results of other studies based on observations of concentration–time changes of PAcBA [11,14], indicate that it is quickly eliminated. Such rapid elimination is likely to increase the Vd_area_/F value, but further studies using single intravenous administration are necessary to assess the actual value of total body clearance (Cl_B_) and Vd_area_.

## 3. Materials and Methods

### 3.1. Animals and Drugs

Nine Polish Large White × Polish Landrace piglets from one litter, with an initial body weight of 58.3 ± 4.09 kg, were used in the experiment. The animals were kept in an experimental animal facility at the Faculty of Veterinary Medicine of the University of Warmia and Mazury in Olsztyn, Poland, in three separate 4 × 4 m stalls (three animals in each) with automatic drinking bowls in which water was available ad libitum. The animal facility was equipped with a forced ventilation system protected by HEPA filters. The facility structure and the installed equipment allowed constant temperature (21 °C), relative humidity (65%) and air flow (0.2 m/s) to be maintained. Granular feed (WIPASZ, Wadąg, Poland) was given to the animals in an amount of 850 g/animal twice a day at 8.00 a.m. and 5.00 p.m., throughout the experiment. The content of nutrients in the feed, as declared by the manufacturer, is presented in Supplementary Table S6. The pigs did not receive any pharmacological treatment during the acclimatization period. The study was registered and approved by the Local Ethics Committee in Olsztyn (Ethics Committee Opinion No. 17/2014).

### 3.2. Chemicals and Reagents

For liquid chromatography–tandem mass spectrometry (LC-MS/MS), water, ACN, FA, and methanol (all LC/MS grade) were purchased from Sigma-Aldrich. Analytical standards for PAcBA and deuterium-labeled PAcBA (for use as an IS) were purchased from Sigma-Aldrich (Darmstadt, Germany) and Toronto Research Chemicals (Toronto, ON, Canada), respectively. The stock solution of 1 mg/mL of PAcBA and 0.2 mg/mL of IS was prepared in methanol in 5 mL volumetric borosilicate flasks supplied by the Duran Group (Mainz, Germany). Next, these solutions were taken to make working solutions that were used during the experiments and validation of the method. They were prepared in 5 mL volumetric borosilicate flasks by diluting stock solutions in methanol at the following concentrations: 0.25, 0.625, 1.25, 2.5, 6.25, 12.5, 25.0, 62.5, 125.0, 187.5, and 250.0 µg/mL. All solutions were refrigerated at 4 °C. The gases required for the LC-MS/MS system were nitrogen from a NitroGen N110R nitrogen generator, which was supplied by Peak Scientific (Inchanian, Scotland, UK), and argon, which was purchased from EUROGAZ-BOMBI (Olsztyn, Poland).

### 3.3. Experimental Design

After one week of acclimatization, the animals were divided into three equal groups (*n* = 3 in each group), in which the drug was administered via drinking water 2 h after morning feeding (water was given ad libitum) at doses of 20, 40, and 80 mg/kg in groups 1, 2, and 3, respectively. Blood samples (2 mL each) were collected into heparinized tubes from the right jugular vein through injection needles (1.2 × 80 mm) at 0, 1.0, 2.0, 3.0, 4.0, 5.0, 6.0, 7.0, 8.0, 9.0, 10.0, and 11.0 h after drug administration. Plasma was separated by centrifugation at 1650× *g* for 10 min at 4 °C and was stored at −81 °C until analysis.

### 3.4. Chromatography

The plasma concentration of PAcBA was determined using LC-MS/MS (Supplementary Figure S6). Drug levels were quantified with a Waters Alliance 2695 reversed-phase liquid chromatography system coupled with a tandem mass spectrometer (MS/MS) Quattro micro API MS (Milford, MA, USA). The analytical column was an Atlantis T3 (150 × 3 mm) with a 3 µm particle size, supplied by Waters. The optimal mobile phase was composed of phase A, water with 0.2% FA, and phase B, ACN with 0.2% FA. The gradient elution was based on the time set on the pump. The injection volume was 10 µL, the column temperature was set at 20 °C, and the flow rate was 0.40 mL/min. PAcBA was monitored from *m/z* 180.20 to 94.0 and IS was monitored from *m/z* 183.20 to 95.0 (Table 1).

### 3.5. Sample Preparation

Plasma obtained from humans and 12 different animals was thawed at room temperature, and 250 µL of each sample was combined with 10 μL of IS (1 μg/mL) and mixed in a vortex at 1000 rpm for 5 s. Next, 1 mL of ACN was added for protein precipitation, and the samples were vortexed at 3000 rpm for 10 s. After centrifugation at 40,000× *g* for 10 min at 4 °C, 150 μL of the supernatant was transferred through a 0.22 μm nylon syringe filter (13 mm in diameter) into chromatographic total recovery vials and injected into the chromatographic system.

### 3.6. Method Validation

The analytical method was fully validated using the analytical method validation protocol of the United States Food and Drug Administration (FDA), the European Medicines Agency (EMA) bioanalytical method validation requirements [12,13], and a tutorial review of liquid chromatography–mass spectrometry method validation [15,16]. 

During the validation procedure, the following parameters were determined: linearity, accuracy, precision (repeatability/intra-day precision and intermediate precision/inter-day precision), LOD, LLOQ, selectivity, recovery, matrix effect (ionization suppression/enhancement), carry-over, and stability (freeze–thaw stability, autosampler stability, working standard, stock stability, and sample processing temperature stability). The acceptance criteria were established based on [12,13,15,16], and they are summarized in Table 2. For validation, plasma from healthy pigs was used, and then an additional test was performed to determine the matrix effect and recovery from plasma obtained from healthy human volunteers and from 12 different animal species.

### 3.7. Linearity

The linearity of the method for assaying PAcBA in plasma by HPLC-MS/MS was determined using an 11-point standard curve (0.01, 0.025, 0.5, 0.1, 0.25, 0.5, 1.0, 2.5, 5.0, 7.5, and 10.0 µg/mL) that was prepared four times at one-day time intervals. Each curve was analyzed twice, and each analysis was preceded by a sample without any analytes (blank sample) and a sample containing only IS (zero sample). The values obtained from this test are summarized in Table 2, including the back-calculated concentration, the slope “a” and the intercept “b” in the equation y = bx + a, the Pearson correlation coefficient “r” (and the coefficient of determination, “r^2^”), and the acceptance criteria.

### 3.8. Precision and Accuracy

Precision and accuracy were determined by preparing analyte concentrations at the four QC points and the LLOQ, which were all within the range of the standard curve; this was done three times (at specified time intervals) in six replicates together with IS, according to the method of drug determination previously established by experiment. The analysis yielded concentrations relative to the declared nominal concentration for each QC, obtained by back calculation, and the acceptance criteria are summarized in Table 2.

### 3.9. Limit of Detection

The limit of detection was determined based on the results for the LLOQ obtained in accordance with the parameters specified in Table 2.

### 3.10. Selectivity/Specificity

To identify endogenous matrix elements that may be present during PAcBA or IS retention (Table 2), an analysis was performed on plasma samples obtained from blood collected from pigs not exposed to drugs and, additionally, from 11 animal species and from humans free from exogenous substances. Each sample was prepared six times using the method developed here. The analysis of each matrix sample was separated by analysis of a sample with only a mobile phase.

### 3.11. Recovery

To estimate the degree/effectiveness of extraction, six replicates of each QC were prepared, in which the analytes were added to the plasma either before or after extraction. For samples that had analytes added after extraction, 10 µL of analyte and 10 µL of IS were added after the extraction of an “empty sample.” The concentrations of the analyte and IS added following the extraction were regarded as 100% recovery (Table 2).

### 3.12. Matrix Effect

PAcBA and IS were added to the phase obtained following extraction of an empty matrix in six replicates for each QC. Next, the signal from each compound was compared to that of PAcBA and IS added to a mixture of water (which replaced plasma) and ACN, which was also prepared in six replicates for each QC. Subsequently, following the method presented here, all 48 samples for each of the tested matrices were analyzed. Signals from the analysis of PAcBA and IS in the mixture of water and ACN were considered to have values of 100% (Table 2).

### 3.13. Carry Over

This test was conducted to eliminate any possible PAcBA and IS carry over (ghost peaks) between samples when using the chromatographic system elements (e.g., injector, column, mobile phase). Six replicates of HQC (with IS) and six blank samples were prepared, and the blank sample was analyzed after each HQC sample analysis (Table 2).

### 3.14. Stability

The stability of PAcBA and IS in the matrix, stock solutions, and working solutions was determined at each stage of sample storage, sample preparation, and chromatographic analysis. The results (as peak areas) were compared with the results obtained with freshly prepared standards (Table 2). Moreover, an analysis of blank samples was always prepared and conducted in each stability test to verify the sample preparation procedure for further test stages.

This test was conducted for the QC working solutions and the stock solutions of PAcBA and IS that were stored in a refrigerator at 2 °C. The test was performed at 72 and 120 h for the working solutions and at 120 h for the stock solutions. These solutions were prepared on the first day and 30 samples (six replicates for each solution) were analyzed without extraction. After 72 h of storage of the working solutions, 24 samples were prepared and analyzed, and after 120 h, 30 samples were prepared and analyzed (working solutions + stock solutions).

This test was also performed to check the sample stability in the autosampler operating at 4 °C after 24 and 48 h. A set of samples of six replicates of each QC (24 samples) was prepared on the first day in accordance with the sample preparation protocol and was subsequently analyzed. A second analysis of the same samples was performed after 24 h, and a third was performed after 48 h.

A test was conducted to determine the stability of the drug and IS in the matrix at the sample storage temperature (−81 °C). For each QC, five sets of samples were prepared with six replicates each. The test was conducted at 0, 24, 48, 96, and 1680 h (the final time was the long-term freeze and thaw stability test). The first set of 24 samples was analyzed immediately after preparation (without freezing—day 0), and the remaining samples were frozen at −81 °C. All of the samples were thawed on the next day, the second set was analyzed, and the remaining samples were refrozen. The same procedure was followed with sets three (two days after day 0) and four (four days after day 0). Set five was stored for 70 days (−81 °C) and then analyzed.

Another test was conducted to determine the stability of the drug and IS in the matrix under the sample preparation conditions. Twenty-four samples (all QCs) were prepared from freshly prepared standard solutions and analyzed. At the same time, drug standards were added to another 24 samples (all QCs) and the sample preparation procedure was stopped for 3 h. After that time, the samples were prepared and analyzed. 

### 3.15. Pharmacokinetics

The PK analysis was performed using noncompartmental analysis with ThothPro™ software (ThothPro, Gdańsk, Poland). The PK analysis determined the area under the curve (AUC_0→t_) according to the linear trapezoidal rule, the area under the first moment of the curve from 0 to t (AUMC_0→t_), the mean residence time from 0 to t (MRT_0→t_), the slope of the elimination phase (k_el_), the half-life in the elimination phase (t_1/2kel_), the apparent volume of distribution (Vd_area_/F), and the total body clearance (Cl_B_/F) without bioavailability correction. The mean absorption time (MAT) and the half-life in the absorption phase (t_1/2kab_) were calculated using one-compartmental analysis according to Gibaldi and Perrier 1982 [17]. The maximum (C_max_) and the last (C_last_) plasma concentrations and the time of C_max_ and C_last_ were determined individually for each animal and were expressed as mean values ± SD. 

## 4. Conclusions

This is the first report on the development and validation of a method that uses HPLC-MS/MS for the quantification of PAcBA in the blood plasma of thirteen species. The results indicate that the method is replicable, precise, accurate, selective, and sensitive. The advantages of the method are its simplicity and effectiveness, as well as the rapidity of sample preparation, which make the method more economical and allow rapid and precise assays of PAcBA in plasma. Although the method uses only protein precipitation for plasma purification (it is relatively “dirty”), it has a high recovery rate, and the matrix effect is small enough to validate the method with proper accuracy and precision. Despite a few other limitations (namely, lower recovery in plasma samples of some species and an LOD that is probably not low enough to estimate potential endogenous PAcBA concentrations), the method is suitable for practical application, as shown by its successful application in a PK study of exogenous PAcBA in pigs after oral Inosiplex administration at three different dosages. The results of the PK study indicate that this compound is rapidly eliminated and that its absorption is not only fast but also increases exponentially depending on the dose.

## Figures and Tables

**Table 1:** Selected liquid chromatography and mass spectrometry parameters.

MS/MS Parameters			Compound	
			PAcBA	PAcBA-d3
Precursor ions (*m/z*)			180.2	183.2
Product ions (*m/z*)			94.0	95.0
Desolvation gas			nitrogen	
Desolvation gas temperature (°C)			350	
Desolvation gas flow (L/h)			800	
Collision gas			argon	
Source temperature (°C)			120	
Gas cell pirani pressure (mbar)			3.24 × 10^−3^	
Electrospray mode			Positive	
Cone voltage (V)			30	
Capillary voltage (kV)			3	
Collision energy (eV)			15	
Dwel (s)			0.200	
Delay (s)			0.010	
Retention time window (min)			6.82–7.35	
**Time** **(min)**	**Mobile Phase (%)**		**Curve**	**Elution (mL/min)**
	A	B		
0.00	99	1	1	0.40
6.00	30	70	6	0.40
7.00	0	100	6	0.40
10.00	0	100	6	0.40
11.50	99	1	6	0.40

PAcBA—4-acetamidobenzoic acid; PAcBA-d3—deuterium labeled (d3) 4-acetamidobenzoic acid (internal standard; IS). A—Phase A: 0.2% formic acid in water. B—Phase B: 0.2% formic acid in acetonitrile.

**Table 2:** Methods of calculation and acceptance criteria for validation parameters.

Parameter		Acceptance Criteria
Linearity	Calibration points	At least 75% of calibration points, but not less than 6, should have a deviation (residual) between nominal and back-calculated concentrations of ±15% or less
	Coefficient of determination (r^2^)	≥0.99
	Relative residuals (*Y_i_*)	$\left|\frac{y_{i}-{\hat{y}}_{i}}{{\hat{y}}_{i}}\right|\times 100\%\leq 20\%$
	SD of Relative residuals (*S_Yi_*)	$\sqrt{\frac{\sum {\left(Y_{i}-\bar{Y}\right)}^{2}}{n-2}}\;\leq 0.1$
Stability	Stock and working standard	$\frac{S_{t}}{S_{0}}\times 100\%=\pm 15\%\;of\;S_{0}$
	Autosampler	
	Freeze and thaw	
	Sample processing temperature	
Precision (RSD or CV)		$\frac{SD}{C_{mean}}\times 100\%=\pm 15\%\;within\;nominal\;concentration$
Accuracy (Deviation) (for at least 5 points per group/day)		$\frac{\left|\left(C_{t}-C_{n}\right)\right|}{C_{n}}\times 100\%=\pm 15\%\;within\;nominal\;concentration$
Limit of detection (LOD)		$3\times SD_{C}{}_{fortified}$ where S/N≥3:1
The lowest limit of quantitation (LLOQ) with accuracy and precision		$6\times C_{fortified}$ where S/N≥10:1
Matrix Effect		$\;100-\left(\frac{X_{i}}{X}\times 100\%\right)\;=\pm 15\%\;compare\;to\;sample\;without\;matrix$
Total Recovery		$\frac{X_{z}}{X_{i}}\times 100\%$ =±15% RSD
Selectivity/Specificity		No endogenous peaks in retention time of analyte
Carry Over		Area of carried peaks ≤20% of LLOQ area, and for IS, 5% of its area

*y_i_*—experimental signal; *ŷ_i_*—calculated signal; *Y_i_*—relative residual; *Ȳ*—mean value of relative residuals; *S_t_*—peak area obtained when analysis is carried out while making a pause with duration *t* in the analysis; *S*_0_—initial peak area determined without introducing any extra pauses in the analysis process (freshly prepared standards); *SD_Cfortified_*—standard deviation calculated from fortified samples with the lowest acceptable concentration; *C_mean_*—mean concentration (ng/mL); *C_n_*—nominal concentration (ng/mL); *C_t_*—calculated individual concentration (ng/mL); *C_fortified_*—minimal fortified concentration that meets requirements; *X*—peak area of analyte in final solvent; *X_i_*—peak area of analyte added to matrix after extraction; *X_z_*—peak area of analyte added to matrix before extraction.

**Table 3:** Selected validation tests results of 4-acetamidobenzoic acid (PAcBA) and deuterium-labeled (d3) 4-acetamidobenzoic acid (internal standard; IS).

**Linearity ^a^**	**r^2^**			**I**	**II**	**III**	**IV**	**Mean**
				0.9992	0.9992	0.9975	0.9988	0.9990
**Precision (%) and accuracy (%)**				**LLOQ**	**LQC**	**IQC**	**MQC**	**HQC**
	Intra-day *n* = 6; 3 repetitions	Precision	I	5.22	3.64	6.17	4.87	2.81
		Accuracy		4.33	2.3	3.91	3.93	2.31
		Precision	II	13.71	11.37	4.45	3.45	4.65
		Accuracy		11.0	8.57	3.61	2.74	3.24
		Precision	III	13.81	5.30	3.10	2.11	3.04
		Accuracy		11.0	4.47	2.38	1.43	2.49
	Inter-day *n* = 18	Precision		10.93	7.08	4.45	3.43	3.38
		Accuracy		8.78	5.11	3.3	2.7	2.68
**LLOQ and LOD ^c^**				**Concentration ^b^**			**S/N**	
	LLOQ overall mean *n* = 18			10.00			15.69	
	LLOQ overall SD *n* = 18			1.09			3.95	
	LOD overall mean *n* = 18			3.27			10.89	
	LOD overall SD *n* = 18			1.48			6.52	
**Carry over**	**Sample**			**Peak Area of PAcBA**		**Peak Area of Mobile Phase**	**Carry Over (%)**	
	**Mean**	**PAcBA**		193,810.4		6.27	4.69	
		**IS**		24,601.28		0	0	
	**SD**	**PAcBA**		2382.238		6.93	5.19	
		**IS**		218.815		0	0	

LQC—low-concentration quality control (50 ng mL^−1^); IQC—intermediate-concentration quality control (500 ng mL^−1^); MQC—medium-concentration quality control (5000 ng mL^−1^); HQC—high-concentration quality control (10,000 ng mL^−1^); LLOQ—the lowest limit of quantitation (nominal concentration 10 ng mL^−1^, mean peak area 133.74); LOD—limit of detection; S/N—signal-to-noise ratio. ^a^ calibration curve range: 10, 25, 50, 100, 250, 500, 1000, 2500, 5000, 7500, and 10,000 ng mL^−1^. ^b^ in ng mL^−1^. ^c^ $\mathrm{LOD}=3\times {\mathrm{SD}}_{\mathrm{LLOQ}\;\mathrm{or}\;S/N_{\mathrm{mean}}}.$

**Table 4:** Stability tests results.

Stability	Period (h)	Compound	Decrease/Increase of Quality Control Concentration (%)			
			LQC	IQC	MQC	HQC
Stock2 °C	120	PAcBA	−8.42	−11.12	−14.59	−12.85
		IS	−8.74	−4.17	−4.99	−10.21
Working standard 2 °C	72	PAcBA	−7.64	7.81	3.72	1.71
		IS	8.31	−2.74	−4.28	−11.71
	120	PAcBA	−14.01	−0.14	0.65	2.80
		IS	−2.21	−8.27	−9.54	−14.88
Autosampler 4 °C	24	PAcBA	2.17	−0.825	0.19	1.05
		IS	1.44	6.01	3.14	1.76
	48	PAcBA	2.03	1.14	1.35	4.31
		IS	0.60	−7.85	−4.09	−4.53
Freeze and thaw −75 °C	24	PAcBA	4.60	−4.33	0.07	4.60
		IS	−5.17	2.04	−4.63	−3.09
	48	PAcBA	4.60	−3.43	−1.77	1.06
		IS	9.58	4.35	5.26	1.22
	96	PAcBA	3.68	−2.02	−2.84	1.54
		IS	0.34	−7.24	−10.10	−11.78
	1680	PAcBA	6.09	−4.80	−1.78	1.14
		IS	−13.06	−6.98	−10.23	−12.21
Sample processing temperature 21 °C	3	PAcBA	−3.36	0.28	−0.14	−0.48
		IS	−5.50	1.01	2.09	1.85

LQC—low-concentration quality control; IQC—intermediate-concentration quality control; MQC—medium-concentration quality control; HQC—high-concentration quality control; PAcBA—4-acetamidobenzoic acid; IS—internal standard.

**Table 5:** Pharmacokinetic parameters (noncompartmental analysis) of 4-acetamidobenzoic acid in pigs after oral administration at different doses.

Pharmacokinetic Parameters	Dose (mg/kg)		
	20	40	80
	Mean ± SD	Mean ± SD	Mean ± SD
AUC_(0→t)_ (μg·h/L)	878.74 ± 372.3 ^a^	3402.52 ± 1687.26 ^a^	12,868.1 ± 4896.6 ^b^
AUMC_(0→t)_ (μg·h·h/L)	5935.97 ± 2453 ^a^	19,015.92 ± 7157.79 ^a^	88,002.9 ± 38,611.43 ^b^
C_max_ (ng/mL)	406.73 ± 211.6 ^a^	2079.87 ± 787.56 ^a^	4272.27 ± 1713 ^b^
t_max_ (h)	6 ± 1	5 ± 1.5	6 ± 1
C_last_ (ng/mL)	12.32 ± 3.89 ^a^	10.21 ± 2.11 ^a^	68.11 ± 25.1 ^b^
t_last_ (h)	11	11	11
k_el_ (h^−1^)	0.49 ± 0.21	0.62 ± 0.31	0.81 ± 0.37
t_1/2kel_ (h)	1.42 ± 0.87	1.12 ± 0.42	0.85 ± 0.29
MRT_(0→t)_ (h)	6.76 ± 3.21	5.59 ± 2.78	6.84 ± 3.41
Cl_B_/F (L·h)	682.8 ± 214.23 ^a^	352.68 ± 131.2 ^a,b^	186.51 ± 97.6 ^b^
Vd_area_/F (L)	1399.78 ± 731.6	568.33 ± 243.65	229.56 ± 112.38
k_ab_ (h^−1^)	0.27 ± 0.11 ^a^	0.81 ± 0.37 ^a^	1.96 ± 0.89 ^b^
t_1/2kab_ (h)	2.57 ± 1.21 ^a^	0.86 ± 0.31 ^a^	0.36 ± 0.13 ^b^
MAT (h)	3.70 ± 1.7 ^a^	1.23 ± 0.41 ^a^	0.51 ± 0.19 ^b^

AUC_0→t_—area under the concentration vs. time curve from 0 to t; AUMC_0→t_—area under the first moment of the curve; C_max_—maximum plasma concentration; t_max_—time of maximum concentration; C_last_—last measured plasma concentration; t_last_—time of last measured concentration; k_el_—elimination rate constant; t_1/2kel_—half-life in elimination phase; MRT_0→t_—mean residence time; Cl_B_/F—total body clearance without bioavailability correction; Vd_area_/F—apparent volume of distribution without bioavailability correction; k_ab_—absorption rate constant; t_1/2kab_—half-life in absorption phase; MAT—mean absorption time. ^a,b,c^ Pharmacokinetic parameters differ significantly (*p <* 0.05) between the groups.

## Data Availability

The data presented in this study are available on reasonable request from the corresponding author.

## Supplementary Materials

The following are available online: Figure S1: Chromatograms from an Atlantis T3 analytical column (150 × 3 mm with 3 µm particle size)—A, A’, and from an XBridge column (150 × 3 mm with 3.5 µm particle size)—B, B’ (A,B—4-acetamidobenzoic acid; A’,B’—deuterium labeled 4-acetamidobenzoic acid as an internal standard). Figure S2: Chromatograms obtained from initial phase comprised of 0.2% formic acid in water with 0.2% formic acid in acetonitrile at a ratio of 9:1 *v*/*v* with the column temperature set at 35 °C (A—unidentified background noise; A’—asymmetrical peak at the top). Figure S3: Chromatograms obtained at the lowest limit of quantitation, 10 ng/mL (A—4-acetamidobenzoic acid; A’—deuterium labeled 4-acetamidobenzoic acid as an internal standard). Figure S4: Chromatograms from a selectivity/specificity test. The signal-to-noise ratios of all identified peaks are lower than 10:1. Figure S5: Power regression with the coefficient of determination (R2) of the area under the concentration-time curve calculated from 0 to t (AUC_0→t_) and logarithmic regression with the coefficient of determination (R2) of the maximum plasma concentration (Cmax) of 4-acetamidobenzoic acid after oral administration to pigs at doses of 20, 40 and 80 mg/kg BW (n = 3). Figure S6: Chromatograms obtained from the pharmacokinetics study: A—4-acetamidobenzoic acid; A’—deuterium labeled 4-acetamidobenzoic acid as an internal standard. Table S1: Matrix effect and total recovery results of PAcBA and IS using three different extractants. Each quality control point is the mean value calculated from six replicates. Table S2: Linearity results. Table S3: Precision and accuracy results. Table S4: The lowest limit of quantitation (LLOQ) and limit of detection (LOD) results. Table S5: Carry over test results of 4-acetamidobenzoic acid (PAcBA) and deuterium labeled (d3) 4-acetamidobenzoic acid (internal standard; IS). Table S6: Feed composition.

## Sample Availability

Not available.
