# Supplementary material for: Development, Validation, and Application of the LC-MS/MS Method for Determination of 4-Acetamidobenzoic Acid in Pharmacokinetic Pilot Studies in Pigs
Source: Molecules. 2021 Jul 23;26(15):4437. doi: 10.3390/molecules26154437 (PMC8348927; doi:10.3390/molecules26154437)
Supplement: Supplementary file 1 [file molecules-26-04437-s001.zip › molecules-1292214-supplementary.pdf]

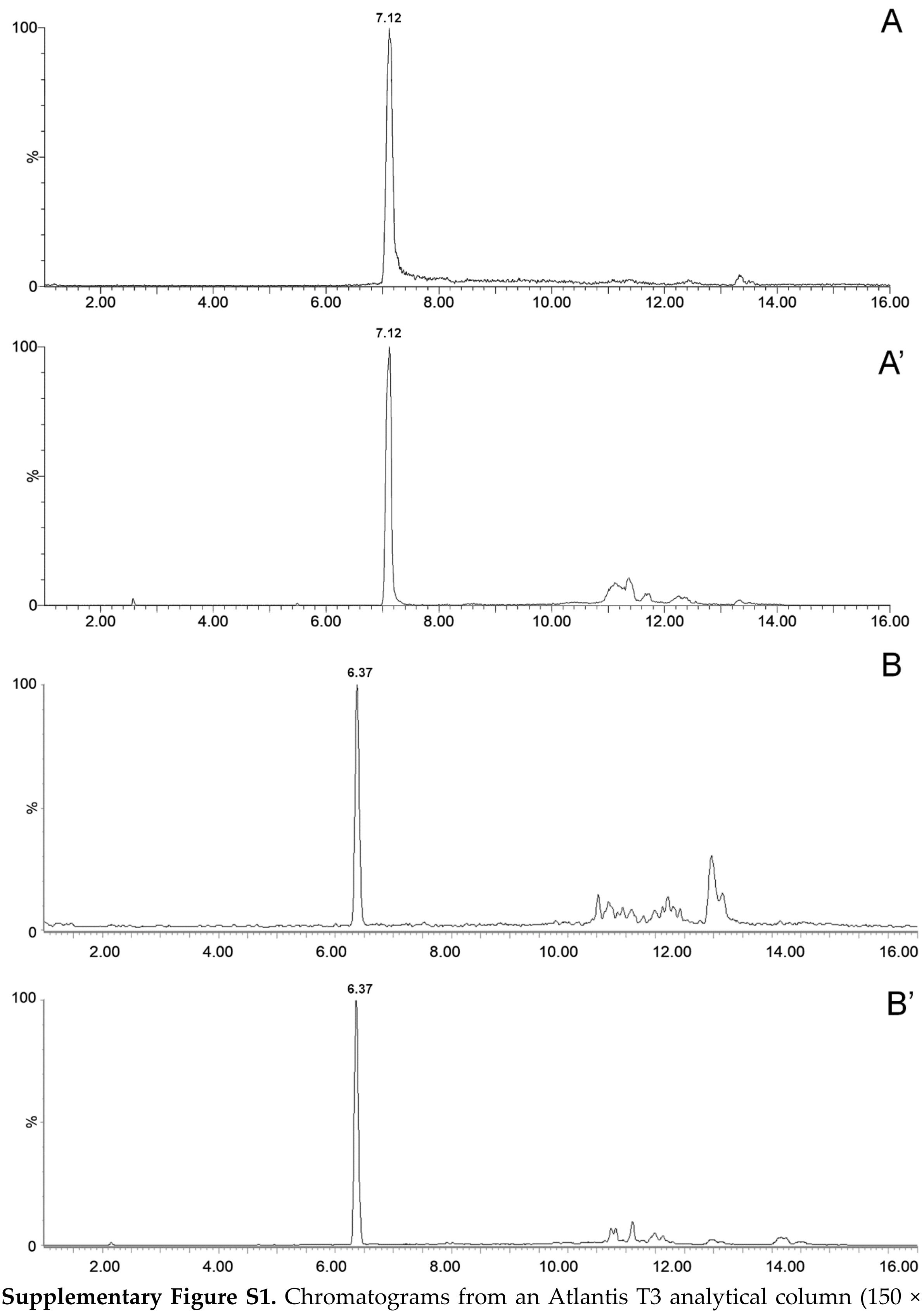

Supplementary Figure S1. Chromatograms from an Atlantis T3 analytical column (150 × 3 mm with 3 µm particle size) – A, A′, and from an XBridge column (150 × 3 mm with 3.5 µm particle size) – B, B′ (A,B – 4-acetamidobenzoic acid; A′,B′ – deuterium labeled 4-acetamidobenzoic acid as an internal standard).

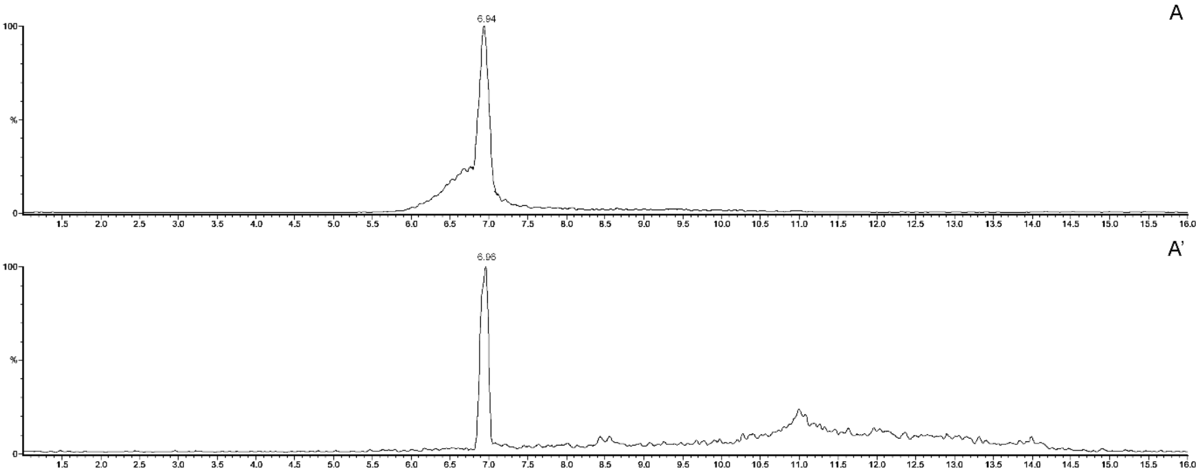

Supplementary Figure S2. Chromatograms obtained from initial phase comprised of 0.2% formic acid in water with 0.2% formic acid in acetonitrile at a ratio of 9:1 v/v with the column temperature set at 35°C (A – unidentified background noise; A′ - asymmetrical peak at the top).

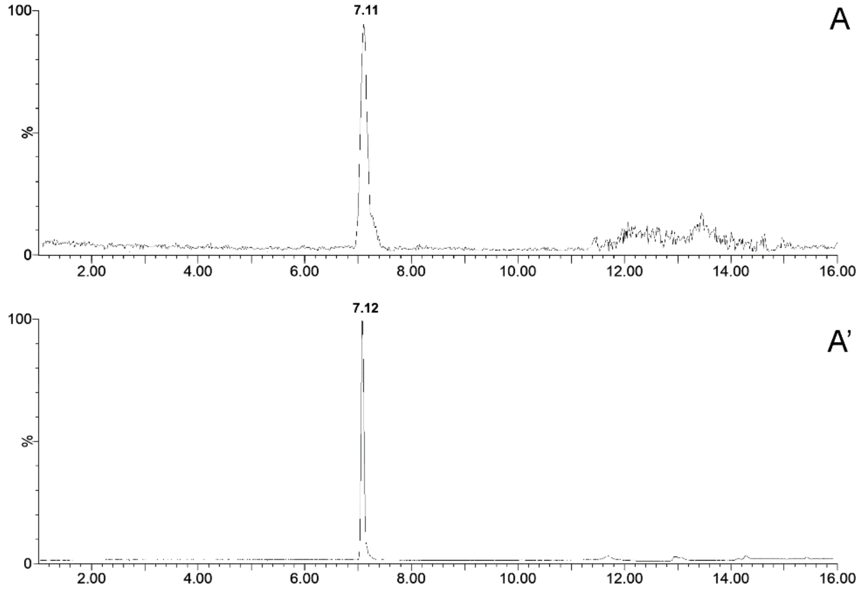

Supplementary Figure S3. Chromatograms obtained at the lowest limit of quantitation, 10 ng/mL (A – 4-acetamidobenzoic acid; A′ – deuterium labeled 4-acetamidobenzoic acid as an internal standard).

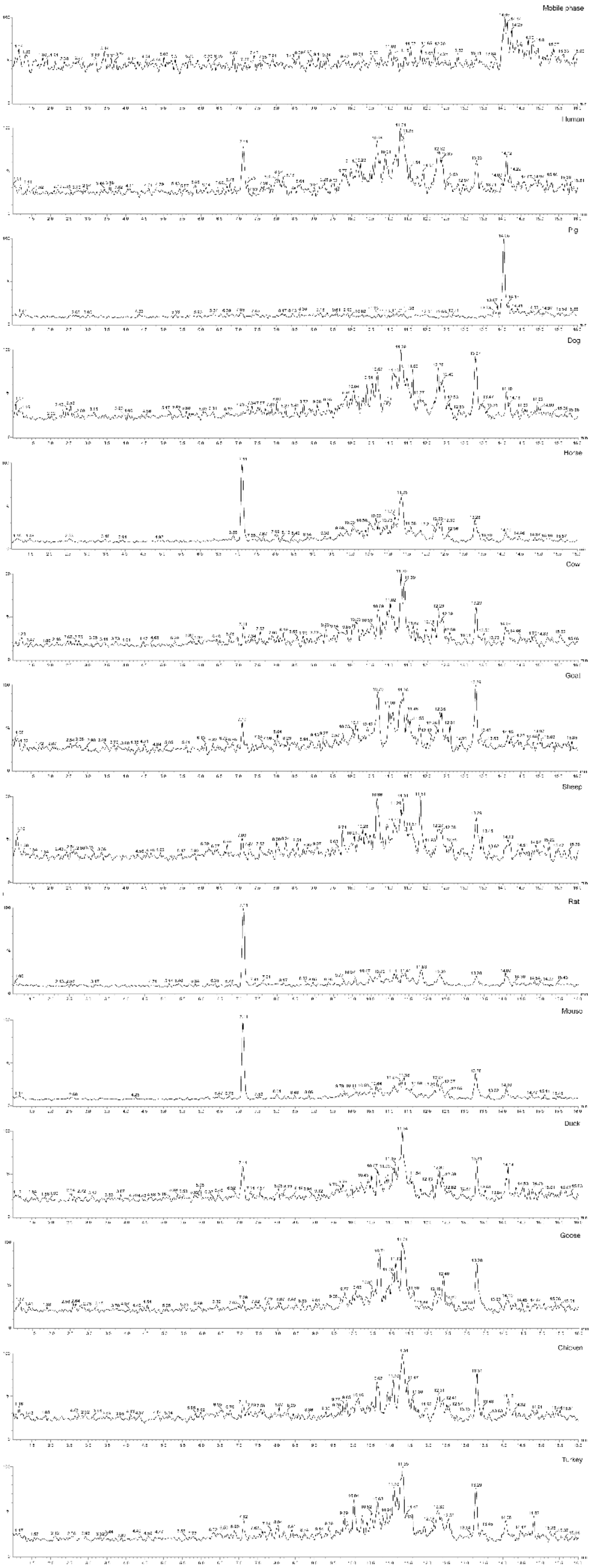
Mobile phase
Human
Pig
Dog
Horse
Cow
Goat
Sheep
Rat
Mouse
Duck
Goose
Chicken
Turkey

Supplementary Figure S4. Chromatograms from a selectivity/specificity test. The signal-to-noise ratios of all identified peaks are lower than 10:1.

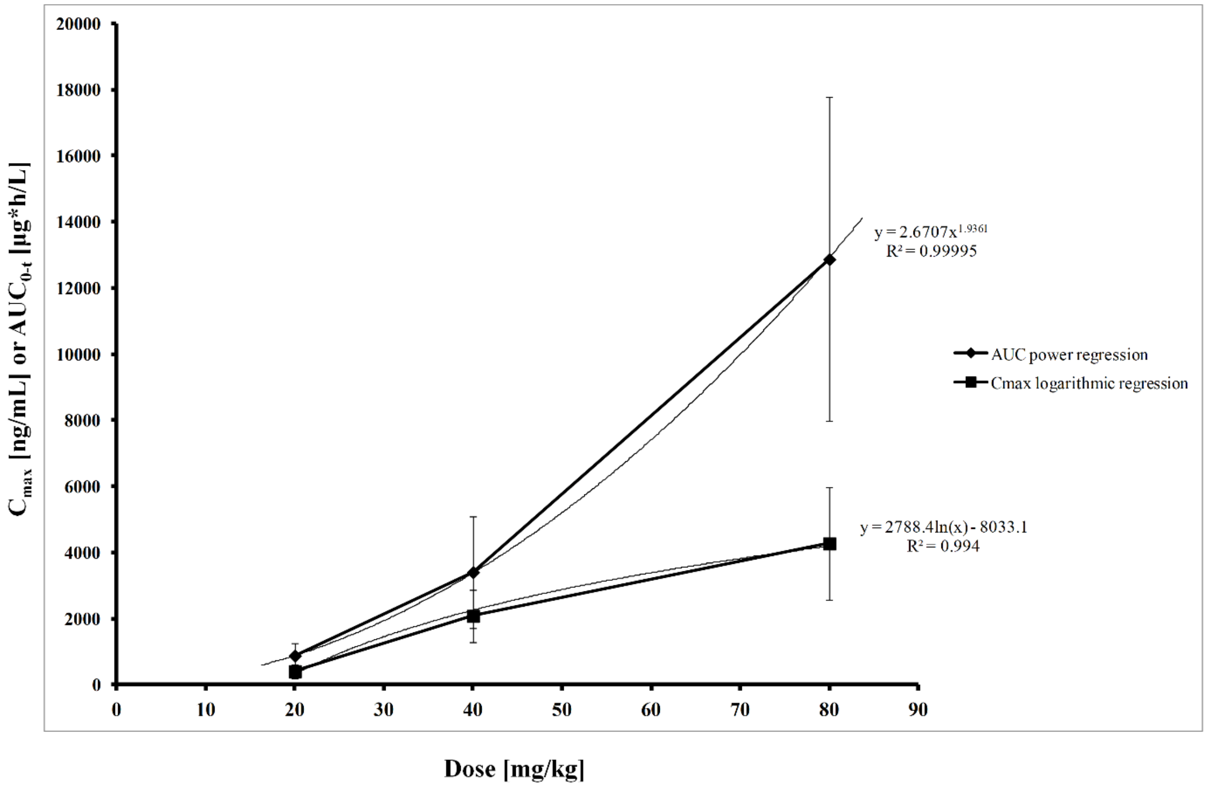

Supplementary Figure S5. Power regression with the coefficient of determination (R^2) of the area under the concentration-time curve calculated from 0 to t ($AUC_{0\to t}$) and logarithmic regression with the coefficient of determination (R^2) of the maximum plasma concentration (C_{max}) of 4-acetamidobenzoic acid after oral administration to pigs at doses of 20, 40 and 80 mg/kg BW (n=3).

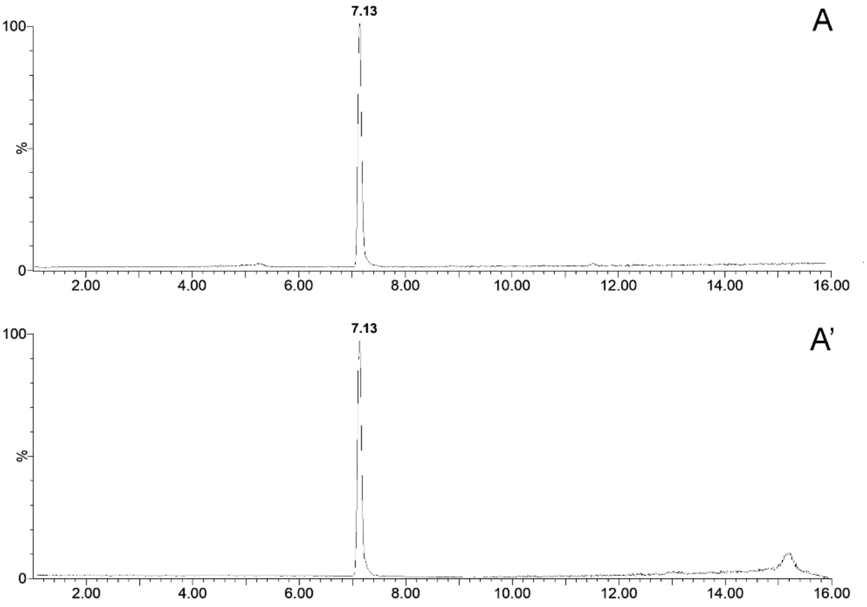

Supplementary Figure S6. Chromatograms obtained from the pharmacokinetics study: A – 4-acetamidobenzoic acid; A′ – deuterium labeled 4-acetamidobenzoic acid as an internal standard.

Supplementary Table S1. Matrix effect and total recovery results of PAcBA and IS using three different extractants. Each quality control point is the mean value calculated from six replicates.

QC	Acetonitrile				1,2 Dichloroethane				Ethyl Acetate			
	Recovery		Matrix effect		Recovery		Matrix effect		Recovery		Matrix effect	
	PAcBA	IS	PAcBA	IS	PAcBA	IS	PAcBA	IS	PAcBA	IS	PAcBA	IS
**LQC**	88.46	84.90	-3.36	-5.95	90.44	93.37	14.96	11.65	94.80	95.19	15.54	12.42
**IQC**	86.27	87.73	-11.29	-5.29	94.42	98.95	13.11	15.93	91.73	90.07	14.14	11.96
**MQC**	88.10	87.55	-1.69	-6.12	90.43	94.44	10.95	11.30	96.49	95.97	11.77	17.80
**HQC**	92.47	92.20	-3.73	-5.44	95.70	98.62	10.55	11.18	98.58	94.98	10.23	11.15
**Mean**	88.83	88.09	-5.02	-5.70	92.75	96.35	12.39	12.52	95.41	94.06	12.92	13.33
**SD**	2.61	3.02	4.27	0.39	2.72	2.85	2.04	2.28	2.89	2.69	2.37	3.02

QC – quality control point; LQC – low concentration quality control; IQC – intermediate concentration quality control; MQC – medium concentration quality control; HQC – high concentration quality control; PAcBA – 4-acetamidobenzoic acid; IS – internal standard (deuterium labeled 4-acetamidobenzoic acid).

Supplementary Table S2. Linearity results.

Linearity parameters	Calibratione curve				
	I	II	III	IV	Mean[a]
Range[b]	10, 25, 50, 100, 250, 500, 1000, 2500, 5000, 7500,10000				
Number of calibration points above 15% deviation	1	2	2	2	2
r^2	0.9992	0.9992	0.9975	0.9988	0.9990
Regression equation	y = 1.0056x + 4.3222	y = 0.9973x + 2.1339	y = 0.9974x + 6.2388	y = 1.0025x + 11.967	y = 1.0007x + 6.1655
Mean relative residuals (%)	6.92	2.69	7.22	11.46	7.53
SD of relative residuals	0.01	0.01	0.01	0.02	0.01

[a] the curve created from mean concentrations.

[b] in ng ml^{-1}.

Supplementary Table S**3**. Precision and accuracy results.

		LLOQ	LQC	IQC	MQC	HQC
	**Nominal concentration[a]**	10.0	50.0	500.0	5000.0	10000.0
Intra-day n=6; 3 repetitions	Mean concentration[a] ±SD	10.0 ± 0.52	49.98 ± 1.82	500.0 ± 30.84	4999.98 ± 243.42	10000.02 ± 281.34
	Precision (%)	5.22	3.64	6.17	4.87	2.81
	Accuracy (%)	95.67	97.7	96.09	96.07	97.69
	Mean concentration[a] ±SD	10.0 ± 1.37	49.98 ± 5.68	500.0 ± 22.34	5000.0 ± 172.25	10000.02 ± 465.29
	Precision (%)	13.71	11.37	4.45	3.45	4.65
	Accuracy (%)	89.0	91.43	96.39	97.26	96.76
	Mean concentration[a] ±SD	10.0 ± 1.38	50.0 ± 2.65	500.02 ± 15.51	5000.02 ± 105.63	10000.0 ± 304.15
	Precision (%)	13.81	5.30	3.10	2.11	3.04
	Accuracy (%)	89.0	95.53	97.62	98.57	97.51
Inter-day n=18	Mean concentration[a] ±SD	10.0 ± 1.09	49.98 ± 3.54	500.01 ± 22.27	5000.0 ± 171.57	10000.01 ± 337.88
	Precision (%)	10.93	7.08	4.45	3.43	3.38
	Accuracy (%)	91.22	94.89	96.7	97.3	97.32

LQC – low-concentration quality control (50 ng ml^{-1}); IQC – intermediate-concentration quality control (500 ng ml^{-1}); MQC – medium-concentration quality control (5000 ng ml^{-1}); HQC – high-concentration quality control (10000 ng ml^{-1}).

[a] in ng ml^{-1}.

Supplementary Table S4. The lowest limit of quantitation (LLOQ) and limit of detection (LOD) results.

LLOQ – the lowest limit of quantitation; LOD – limit of detection; S/N – signal to noise ratio.

PAcBA nominal concetration[a]	No.	Repetition					
		I		II		III	
		concentration[a]	S/N	concentration[a]	S/N	concentration[a]	S/N
10.0	1	10.60	8.35	8.50	15.17	9.20	11.91
	2	9.30	16.74	8.90	12.56	8.60	17.66
	3	9.50	13.36	10.60	11.18	12.30	22.23
	4	10.40	17.18	10.50	14.35	9.60	17.14
	5	9.90	13.32	9.30	11.92	9.30	22.95
	6	10.30	15.86	12.20	11.33	11.00	29.21
Mean		10.00	14.13	10.00	12.75	10.00	20.18
SD		0.52	3.28	1.37	1.65	1.38	5.96
LOD[b]		1.56	9.84	4.11	4.96	4.14	17.87

	concentration[a]	S/N
LLOQ overall mean n=18	10.00	15.69
LLOQ overall SD n=18	1.09	3.95
LOD overall mean n=18	3.27	10.89
LOD overall SD n=18	1.48	6.52

[a] ng ml^{-1}

[b] $LOD = 3 \times SD_{LLOQ\ or\ S/N_{mean}}$

Supplementary Table S5. Carry over test results of 4-acetamidobenzoic acid (PAcBA) and deuterium labeled (d3) 4-acetamidobenzoic acid (internal standard; IS).

Sample		Peak area of PAcBA	Peak area of mobile phase	Peak area of PAcBA LLOQ	Carry over (%)
1	PAcBA	192167.07	0	133.74	0
	IS	24805.0	0		0
2	PAcBA	197217.39	12.42		9.29
	IS	24245.05	0		0
3	PAcBA	195457.67	0		0
	IS	24488.53	0		0
4	PAcBA	192124.78	0		0
	IS	24575.0	0		0
5	PAcBA	191073.66	14.09		10.54
	IS	24660.18	0		0
6	PAcBA	194821.64	11.12		8.32
	IS	24833.93	0		0
Mean	PAcBA	193810.4	6.27	-	4.69
	IS	24601.28	0	-	0
SD	PAcBA	2382.238	6.93	-	5.19
	IS	218.815	0	-	0

LLOQ – the lowest limit of quantitation.

Supplementary Table S6. Feed composition.

**Basic composition**	**Unit**	**Contents**
Crude protein	%	16.6
Crude fat	%	3.7
Crude fiber	%	4.6
Lysine	%	1.06
Methionine	%	0.33
Treonine	%	0.69
Tryptophan	%	0.18
Calcium	g/kg	5.6
Phosphorus	g/kg	4.3
Sodium	g/kg	1.9
Vit. A	UI	6500
Vit D_3	UI	2000
Vit E	UI	65
